# Comparative Analysis of Transcriptomes of Diploid and Tetraploid *Miscanthus lutarioriparius* under Drought Stress

**DOI:** 10.3390/genes13050873

**Published:** 2022-05-13

**Authors:** Xitong Xu, Shukai Wang, Yanbin Han, Yancui Wang, Pingping Xu, Cuixia Chen, Guobin Zhang

**Affiliations:** 1 State Key Laboratory of Crop Biology, Shandong Agricultural University, Taian 271018, China; xxt_sdjn@163.com (X.X.); sdauwangshukai@163.com (S.W.); hanyanbin1996@163.com (Y.H.); wyc5515@163.com (Y.W.); 18706388706@163.com (P.X.); cxchen@sdau.edu.cn (C.C.); 2 College of Agronomy, Shandong Agricultural University, Taian 271018, China

**Keywords:** *Miscanthus lutarioriparius*, diploid, tetraploid, differentially expressed genes, drought-tolerant

## Abstract

*Miscanthus lutarioriparius* is a species of bioenergy crop unique to China. It is widely distributed in the south of China with high resistance to drought and salt stress. To date, the molecular mechanism of the adaption to drought stress in *M. lutarioriparius* is little known. In this study, RNA-seq technology was employed to analyze the transcriptome changes of diploid and tetraploid *M. lutarioriparius* after drought treatment. It was found that the number of differentially expressed genes in diploid *M. lutarioriparius* was much higher than tetraploid, whereas the tetraploid *M. lutarioriparius* may require fewer transcriptional changes. While the transcriptional changes in drought-tolerant tetraploid *M. lutarioriparius* are less than that of diploid, more known drought-tolerant pathways were significantly enriched than drought-sensitive diploid *M. lutarioriparius*. In addition, many drought-tolerance-related genes were constitutively and highly expressed in tetraploid under either normal condition or drought stress. These results together demonstrated that drought-tolerant tetraploid *M. lutarioriparius*, on the one hand, may preadapt to drought by constitutively overexpressing a series of drought-tolerant genes and, on the other hand, may adapt to drought by actively inducing other drought-tolerant-related pathways. Overall, this study could deepen our understanding of the molecular mechanism of drought-tolerance in bioenergy plants.

## 1. Introduction

Drought stress is one of the main abiotic restricting factors affecting crop production in the world. It is estimated that nearly 50% of reduction in agricultural production is due to drought and other abiotic stresses [1,2]. Long period and high intensity of drought stress not only delays the growth of plants, but also results in changes of the morphological structure and the physiological processes, or even death [3,4]. In order to resist drought stress, plants maintain basic life activities through specific cellular and molecular activities, including the expression of drought-tolerant genes and the accumulation of drought-related proteins [5,6,7]. Therefore, a comprehensive study of the resistance mechanism of plants to drought stress is very important for the genetic improvement of plants’ drought resistance.

Plants exhibit multiple responses to drought stress, including changes in physiological, biochemical, and molecular processes, so as to maintain basic physiological activity [8,9]. When plants are subjected to drought stress, some osmotic regulators (amino acids or sugars) are accumulated in plant cells to prevent water loss [10,11,12]. In the meantime, aquaporins (AQP) can promote water absorption and transmembrane flow to regulate the water balance of the whole plant [13,14,15]. It has been shown that the responses of plants to drought stress are complex processes including multigene and multi-component signaling pathways. In these processes, stress and activated transcription factors (TFs) can activate the expression of drought resistant genes, which lead to change the protein levels of a large number of genes with different functions [16,17]. The newly synthesized proteins then directly or indirectly participate in maintaining the balance of osmolytes, ions, and redox substance [18]. It is obvious that TFs and their signaling pathways play an indispensable role in these processes. TFs such as NAC and MYB [18,19,20], as well as plant hormone signaling pathways and reactive oxygen species (ROS) are advancing research progress about the underlying molecular mechanisms of drought tolerance in a variety of crops, but the research on bioenergy plants is very limited.

*Miscanthus*, characterized by its perennial growth habit and extensive root system as well as high water and nutrient use efficiency, is a promising crop for bioenergy production [20,21,22,23,24,25]. Therefore, Miscanthus has been widely studied. It has been well known that polyploidy plants possess the advantage of higher tolerance to abiotic and biotic stresses than diploid plants [26]. The tetraploid rice derived from colchicine treatment has more biomass and stronger resistance to stresses than diploid rice [27,28,29]. The study of autotetraploid Arabidopsis suggested that the doubling of chromosomes in Arabidopsis promoted the adaptability to high salt [30]. In addition, tetraploids of diverse plant species have stronger drought tolerance than their diploids [31,32,33]. However, most studies of *Miscanthus* were focused on the utilization of biomass and cellulose/hemicellulose, but the difference of tolerance to abiotic stresses between tetraploid and diploid *Miscanthus* and the underlying mechanism for their coping with drought were largely unknown.

*M. lutarioriparius*, with different ploidy level of diploid and tetraploid, is a species exclusively distributed in China. Although this species has relatively high drought tolerance, the growth of it was still inhibited under severe drought conditions.

Nevertheless, it was observed that tetraploid *M. lutarioriparius* showed more robust growth than diploid when water is limited in the field. In this study, the transcriptomes of diploid and tetraploid *M. lutarioriparius* under drought-treated and normal-watered conditions were analyzed and compared. Then, the strategies for tolerating drought stress of *M. lutarioriparius* with different ploidy levels, and the expression patterns of known drought resistance genes under drought treatment, were investigated to explore the underlying molecular mechanism of their differential drought response.

## 2. Material and Methods *M. lutarioriparius*

### 2.1. Plant Materials and Drought Stress Conditions

Two *M. lutarioriparius* accessions were used for the study. M313 was kindly provided by Xin Ai (Hunan Agricultural University, China), and M016 was collected from Hunan province in China (113° E, 28.2° N). The chromosome number for two accessions were 2n = 2x = 38 (M313, diploid) and 2n = 4x = 76 (M016, tetraploid). The subterraneous stems of *M. lutarioriparius* with different ploidy level were separated into single buds and planted in plastic pots with soil matrix. The second leaf *M. lutarioriparius* seedlings were transferred to perlite in the greenhouse with 16 h light/8 h dark cycle, a mean day/night temperature of 25 °C/22 °C and humidity of 55%. Then, the seedlings were irrigated with 200 mL 1/2 Hoagland solution every three days. At around three-leaf age, the irrigation of drought treatment groups was stopped, but the control groups were irrigated as previously. After 8 days of treatment, the leaves and roots of seedlings were separately sampled for RNA-seq. The control groups and the drought treatment groups of the two accessions were set up for two biological replicates. After 28 days treatment, the phenotypes of two accessions were investigated.

### 2.2. RNA Extraction, cDNA Library Preparation, and RNA-seq

Total RNA of each sample was extracted with Trizol reagent. The total RNA of each sample was quantified and verified by NanoDrop 2000c and 0.8% agarose gel electrophoresis. Libraries were prepared following the recommended Illumina Library Preparation protocol. The quality of the libraries was successively detected by Qubit 2.0 Flurometer (Life Technologies, Carlsbad, CA, USA), 3% agarose gel electrophoresis and Agilent Bioanalyzer 2100 system. The cDNA libraries were sequenced using a 2 × 150 bp paired end configuration, and the length of the library is around 300–500 bp. Sequencing was carried out using the HiSeq 4000 systems from Illumina.

### 2.3. De Novo Assembly of Transcripts

Raw reads were initially filtered by using Trimmomatic to remove the adaptor sequences and the low-quality reads [34]. Subsequently, the clean reads from the control groups and drought treatment groups with biological repeats of leaves and roots were combined, and de novo transcriptome assembly was performed by Trinity [35]. RSEM was used for accurate transcript quantification of combined data [36], and the transcript abundance of each unigene was calculated as TPM. Raw assembled transcripts were filtered by removing sequences with a TPM value less than one, and redundant sequences with similarity greater than 90% were filtered out. Finally, two transcriptomes corresponding with diploid and tetraploid were separately obtained as reference transcriptomes for subsequent analyses.

### 2.4. Analysis of Differential Gene Expression

The clean data of each sample were mapped to the reference transcriptome to obtain the TPM of each gene. The expression levels of each gene in the control groups and drought treatment groups were compared, and the DEGs of *M. lutarioriparius* with different ploidy level were screened according to the differential significance criteria (|log2FC| ≥ 1 and FDR < 0.01). The orthologous transcripts between diploid and tetraploid were obtained by aligning the nucleotide sequences of the two reference transcriptomes [37].

### 2.5. GO Functional Annotation and KEGG Enrichment Analysis

The gene annotation of each transcript was obtained by aligning unigenes with the UniProt database (http://www.uniprot.org/ accessed on 21 December 2020) through local BLAST, and the alignment results were loaded into the Trinotate.sqlite database to find the GO annotation. Bingo (https://github.com/CJ-Chen/TBtools/releases, accessed on 24 December 2020) was used to identify GO terms that annotate a list of enriched genes with significant *p*-value of less than 0.005. KO annotations were obtained through KAAS (KEGG Automatic Annotation Server), a visualization tool provided by KEGG official website (http://www.genome.jp/tools/kaas/ accessed on 5 January 2021). After that, we used the OmicShare cloud platform to identify the KEGG pathway enriched by DEGs, and used p-value < 0.05 as the threshold to screen the metabolic pathways in either diploids or tetraploids under drought stress.

## 3. Results

### 3.1. Phenotypes of Diploid and Tetraploid M. lutarioriparius under Drought Stress

To investigate the effect of drought stress on *M. lutarioriparius* with different ploidy levels, well-cultured *M. lutarioriparius* seedlings were further grown without water for 28 days. After long-time drought treatment, the shoots of diploid (M313) and tetraploid (M016) *M. lutarioriparius* wilted, but the degree of wilting of M016 was less (Figure S1B) than that of M313 (Figure S1A). The growth of lateral roots of both *M. lutarioriparius* accessions were inhibited after drought treatment, but the inhibition degree of M313 was greater than that of M016 (Figure S1C,D). These observations showed that the tetraploid *M. lutarioriparius* was more tolerant to drought stress than the diploid.

### 3.2. De Novo Assembly and Evaluation of Transcriptomes

In order to explore the underlying mechanisms of the different drought tolerance of *M. lutarioriparius* with different ploidy levels, RNA-seq based on next generation sequencing (NGS) was applied. Two transcriptomes corresponding with diploid and tetraploid *M. lutarioriparius* were separately obtained through de novo transcriptome assembly (See Materials and Methods). A total of 95,974 and 84,329 unigenes were obtained for diploid (M313) and tetraploid (M016), respectively. The N50 of all unigenes was 1211 bp (M016) and 1092 bp (M313), and the average GC content of them was 49.46% (Table 1). We used Bowtie2 to map the clean reads of each sample to the unigenes, and the average mapping ratio of all samples was 89% (Table 1). After that, we analyzed the unigenes length distribution of diploid and tetraploid *M. lutarioriparius* assembly results, and the ratio of sequence more than 600 bp was 37.41% and 26.22%, respectively (Figure S2). The results showed that both transcriptomes were qualified as reference transcriptomes.

The transcript abundance of each gene in each sample were then estimated as TPM (Transcripts Per Kilobase Million). Pearson correlation coefficient ® of gene expression level of two biological repeats in the treatment group was calculated. Additionally, the r values of leaves and roots of both accessions were greater than 0.8 and 0.7, respectively (Figure S3). These values were indicative of good biological reproducibility of our datasets, which were suitable for subsequent analyses.

### 3.3. Screening of Differentially Expressed Genes

**Figure 1:**
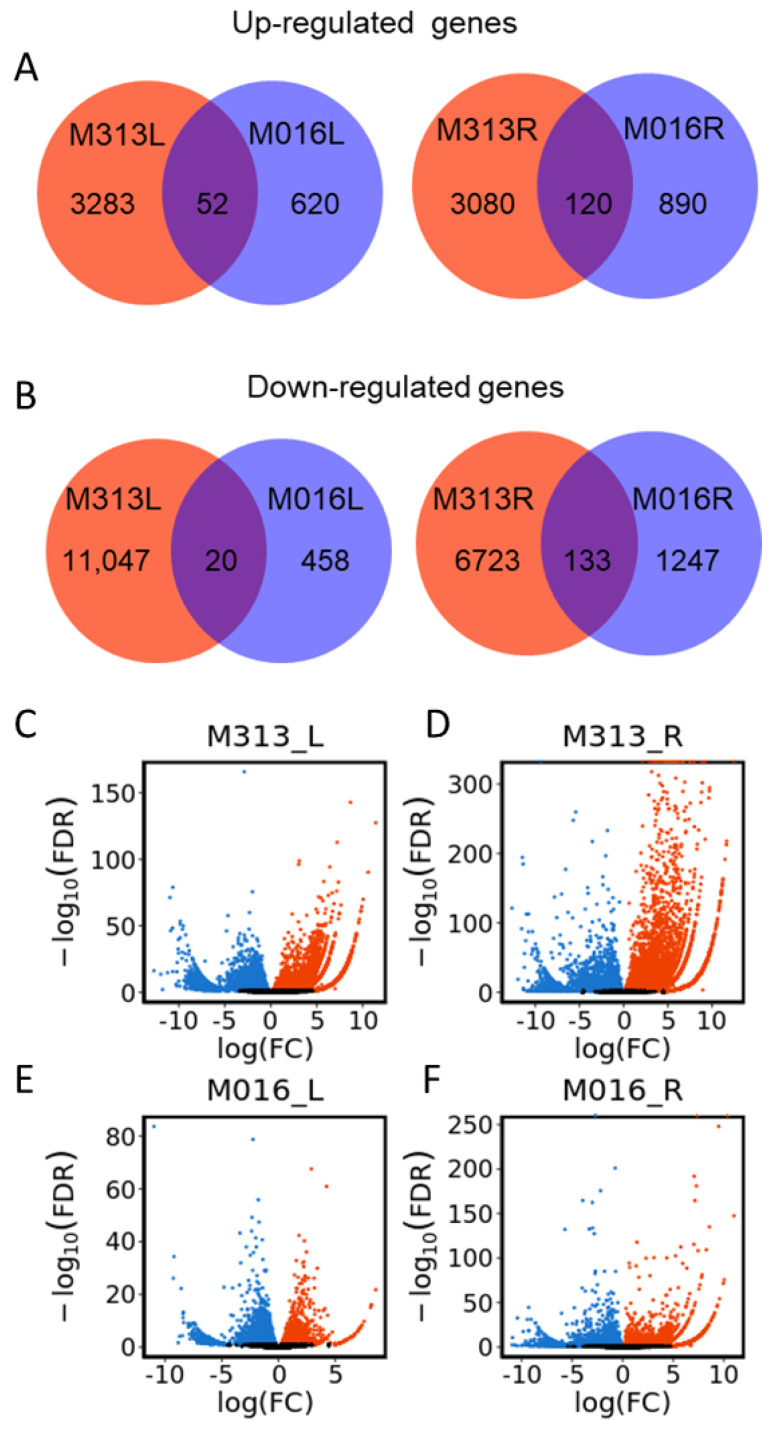
Analysis of DEGs in leaves and roots of *M. lutarioriparius* with different ploidy levels under drought stress. (**A**,**B**) Venn diagram of up-regulated (**A**) and down-regulated (**B**) genes in leaves and roots of *M. lutarioriparius* with different ploidy level. Red circle represents diploid (M313) and blue circle represents tetraploid (M016), L and R represent leaves and roots, respectively. (**C**,**D**) The volcano plot of the DEGs in the leaves (**C**) and roots (**D**) of diploid. (**E**,**F**) The volcano plot of the DEGs in the leaves (**E**) and roots (**F**) of tetraploid. Blue dots represent down-regulated genes, the red dots represent up-regulated genes, and the black dots represent genes with no difference between drought treatment and control group.

To identify differentially expressed genes (DEGs) in *M. lutarioriparius* under drought stress, we used |log2FC| ≥1 and FDR < 0.01 as the threshold to obtain the DEGs in either diploid or tetraploid. There were 14,402 and 10,055 DEGs in leaves and roots of diploid (M313) after drought treatment, respectively (Figure 1A,B). Among them, 3335 genes were up-regulated and 11,067 genes were down-regulated in leaves, whereas 3200 genes were up-regulated and 6855 genes were down-regulated in roots (Figure 1A,B). Meanwhile, 1150 and 2390 DEGs in leaves and roots of M016 were also identified. There were 672 up-regulated genes and 478 down-regulated genes in leaves, whereas 1010 up-regulated genes and 1380 down-regulated genes were identified in roots of tetraploid (M016) (Figure 1A,B). These results showed that the number of DEGs in diploid was much larger than in tetraploid upon drought treatment. Based on these screening results, the gene fold changes in leaves (Figure 1C,E) and roots (Figure 1D,F) of two accessions were shown by volcano plot, which further suggested that there were more up-regulated and down-regulated genes in diploids than tetraploids.

Moreover, 17,757 pairs of orthologous transcripts between diploid and tetraploid *M. lutarioriparius* were obtained by aligning the nucleotide sequences of the two reference transcriptomes. Among them, only 52 up-regulated transcripts and 20 down-regulated transcripts were shared in leaves of both accessions, whereas 120 up-regulated transcripts and 133 down-regulated transcripts were found in their roots (Figure 1A,B).

### 3.4. Analysis of Orthologous DEGs in Response to Drought Stress

**Figure 2:**
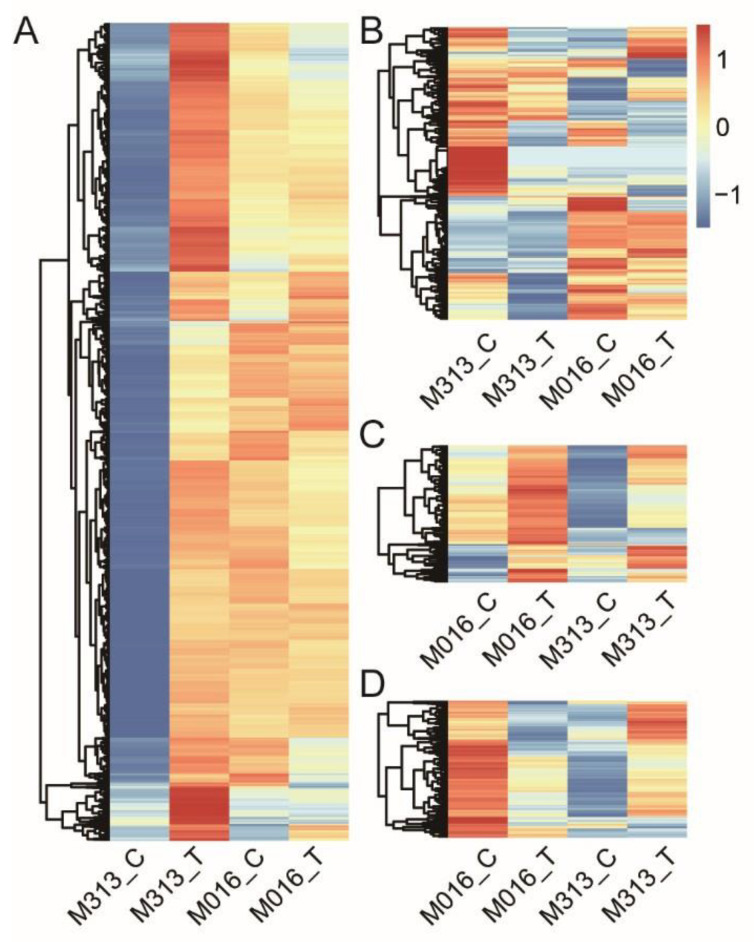
Heatmap of DEGs with orthologous transcripts in the leaves of diploid and tetraploid *M. lutarioriparius*. (**A**) Heat map of up-regulated transcripts with orthologs in leaves of M313. (**B**) Heatmap of down-regulated transcripts with orthologs in leaves of M313. (**C**) Heatmap of up-regulated transcripts with orthologs in leaves of M016. (**D**) Heat map of down-regulated transcripts with orthologs in leaves of M016. Red and blue colors represent up-regulated and down-regulated transcripts, respectively. The darker the color is, the greater the difference of genes expression level is.

As only a small subset of DEGs under drought stress were shared by two *M. lutarioriparius* accessions with different ploidy, it was speculated that two accessions may respond to drought stress in different underlying mechanisms. Thus, we combined all the DEGs identified from M313 and M016 and selected the DEGs with orthologous transcripts (Table S1) shared by diploid and tetraploid *M. lutarioriparius* for further analyses. Among 1840 up-regulated transcripts with orthologs in leaves of M313, most of their corresponding orthologs in M016 were highly and steadily expressed in leaves of either control or treatment group (Figure 2A). Additionally, among the down-regulated transcripts with orthologs in leaves of M313, the gene expression level of most of their corresponding orthologs in leaves of M016 were not evidently changed (Figure 2B). In contrast, the expression levels of 244 up-regulated transcripts in leaves of M016 were also increased in leaves M313 (Figure 2C). Furthermore, the expression pattern of DEGs in roots of both accessions were also similar to that in leaves (Figure S4). These results suggested that stronger transcriptional changes are required for coping with drought stress in diploid *M. lutarioriparius* than tetraploid and a large fraction of transcriptional changes in diploid *M. lutarioriparius* are merely involuntary or passive response upon drought treatment.

### 3.5. Functional Annotation of DEGs

**Figure 3:**
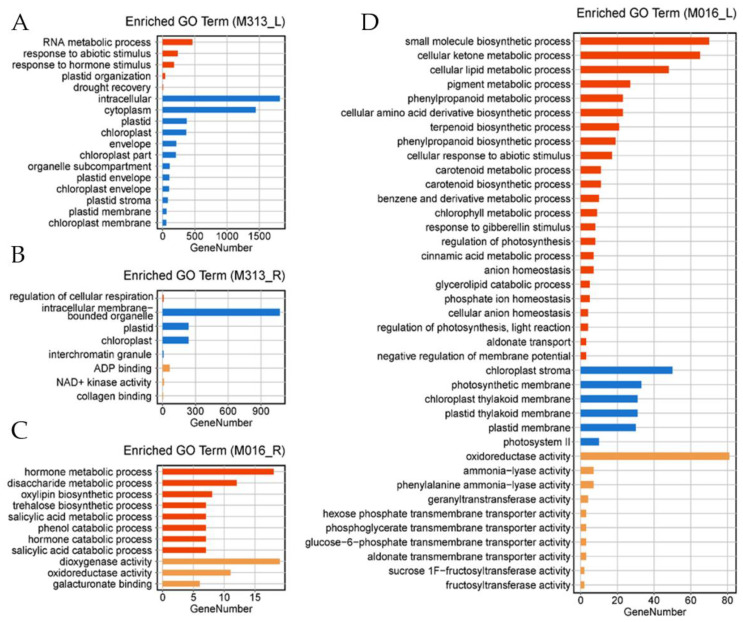
Histogram of GO (Gene Ontology) annotation of up-regulated genes in diploid and tetraploid *M. lutarioriparius* under drought treatment. (**A**) Enriched GO terms in leaves of M313. (**B**) Enriched GO terms in roots of M313. (**C**) Enriched GO terms in roots of M016. (**D**) Enriched GO terms in leaves of M016.

Gene ontology (GO) term enrichment analyses were then performed to classify the DEGs in diploid or tetraploid *M. lutarioriparius*. The enriched GO terms in both leaves and roots of M313 were organ development, RNA metabolic process, photosynthesis, respiration, and other biological processes (Figure 3A,B), most of which were physiological and energy metabolism processes in cells. Only one GO term related to “drought recovery” was identified in the leaves of M313, suggestive of few active biological processes in diploid *M. lutarioriparius* in response to drought.

In the roots (Figure 3C) and leaves (Figure 3D) of M016, the DEGs were mostly enriched in phenylpropanoid biosynthetic process, carotenoid biosynthetic process, glycerolipid catabolic process, hormone metabolic process, fructosyl transferase activity, trehalose biosynthetic process, dioxygenase activity, oxidoreductase activity, etc. These GO terms are mainly related to membrane permeability, biosynthesis of osmotic protectants, scavenging of reactive oxygen species, and signal transduction of plant hormones, which are regarded as active biological processes coping with drought stress.

### 3.6. Enriched Differential Metabolic Pathways in Response to Drought Stress

**Figure 4:**
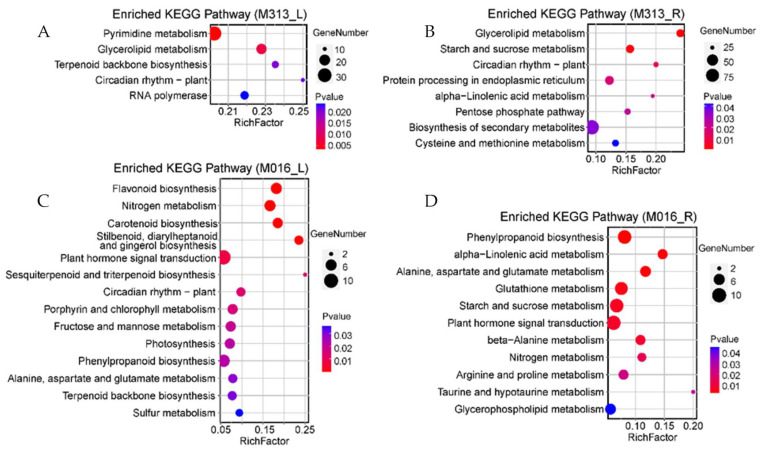
KEGG enrichment pathways of up-regulated genes in leaves and roots of diploid and tetraploid *M. lutarioriparius*. (**A**) Pathway enrichment in leaves of M313. (**B**) Pathway enrichment in roots of M313. (**C**) Pathway enrichment in leaves of M016. (**D**) Pathway enrichment in roots of M016. The color of the dots represents p-value, and the size of the dots represents the number of enriched genes.

To further study the molecular mechanism of differential drought tolerance of two accessions of *M. lutarioriparius* with different ploidy level, the metabolic pathways related to drought tolerance were analyzed by KEGG enrichment. The up-regulated DEGs in the leaves or roots of M313 were mostly enriched in the pathways of the pyrimidine metabolism, RNA polymerase, glycerolipid metabolism, starch and sucrose metabolism, protein processing in endoplasmic reticulum, and pentose phosphate (Figure 4A,B). These pathways are mainly related with metabolism of primary metabolites, which indicated that diploid *M. lutarioriparius* were severely stressed upon drought treatment, and its primary metabolism were greatly perturbed.

The up-regulated DEGs in the leaves and roots of M016, however, were not only enriched in primary metabolism pathways such as amylose metabolism, lipid metabolism, photosynthesis, amino acid metabolism and protein processing, but also enriched in secondary metabolic processes such as the terpenoid backbone biosynthesis, glutathione metabolism, flavonoid biosynthesis, phenylpropanoid biosynthesis, and carotenoid biosynthesis pathways, as well as the plant hormone signal transduction pathway (Figure 4C,D). The enrichment of pathways suggested that tetraploid *M. lutarioriparius* might cope with drought stress through the activation of some secondary metabolites, although its primary metabolism was more or less perturbed.

### 3.7. Enrichment of Induced Genes in Drought Tolerance Pathway

**Figure 5:**
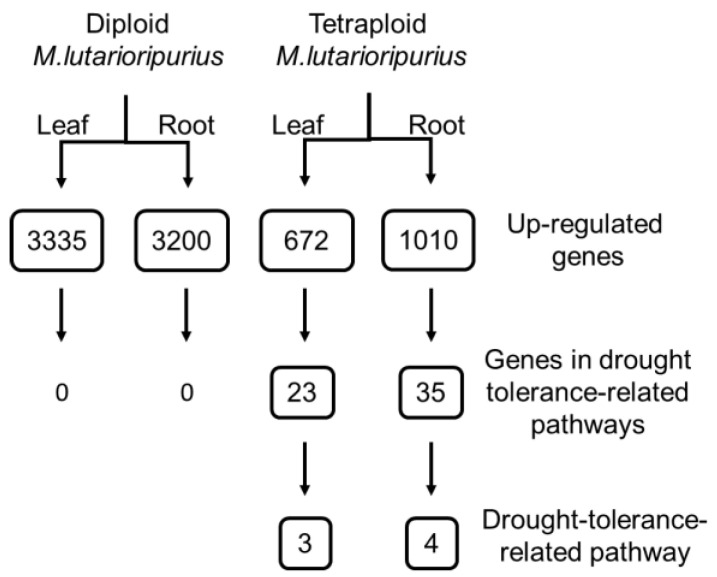
Overview of the DEGs that are enriched in drought-tolerance-related KEGG pathways. The up-regulated genes represent the total up-regulated genes in the leaves and roots of diploid and tetraploid *M. lutarioriparius*, and the genes that were enriched in drought-tolerance-related KEGG pathways were retrieved.

Specifically, the DEGs that were enriched in drought-tolerance-related KEGG pathways were retrieved. In the leaves of M016, 672 genes were up-regulated, of which 23 genes were enriched in three drought-responsive pathways, and 1010 genes in the roots were up-regulated, of which 35 genes were enriched in four pathways related to drought tolerance (Figure 5). However, although there are many more genes induced in the leaves and roots of M313 (Figure S5), no one drought-tolerance-related pathway was enriched (Figure 5). Therefore, although less genes were induced in the tetraploid *M. lutarioriparius*, but more drought-tolerance-related pathways were activated.

### 3.8. DEGs Involved in Key Drought-Tolerance-Related Biological Process

A subset of genes involved in key drought resistance pathways of plants has been elucidated. To explore their roles in the drought tolerance of *M. lutarioriparius* with different ploidy level, their gene expression patterns were analyzed. These genes are involved in the ROS scavenging, biosynthesis of osmolytes, water transport, and some stress-related transcription factors. Most of these genes were down-regulated in M313 roots. However, in the roots of M016, there was no obvious change in the expression of some genes (Figure S6). It thus seems that the expression of these drought-related genes in roots were not responsible for the drought tolerance in either diploid or tetraploid *M. lutarioriparius.*

**Figure 6:**
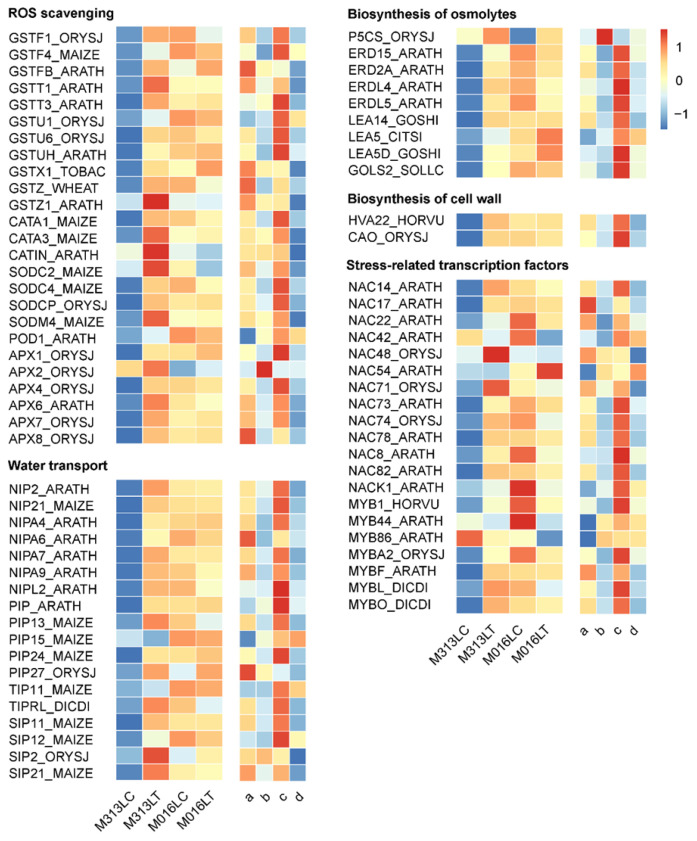
Heatmap of drought-tolerance-related genes in the leaves of diploid and tetraploid *M. lutarioriparius*. Red and blue colors represent up-regulated and down-regulated transcripts, respectively. The darker the color is, the greater the difference of genes expression level is. a, M313T vs. M313C; b, M016T vs. M016C; c, M016C vs. M313C; and d, M016T vs. M313T.

However, these drought-related genes were generally up-regulated in leaves of M313 and consistently highly expressed in leaves of M016 (Figure 6). Notably, even under normal growth conditions, the expression levels of all these drought-related genes were generally higher in leaves of M016 than those in leaves of M313 (Figure 6). These results suggested that tetraploid *M. lutarioriparius* might achieve its strong drought tolerance through sustaining high expression levels of the majority of drought-tolerance-related genes under either normal conditions or drought stress conditions. By contrast, diploid *M. lutarioriparius* might alleviate drought stress through inducing the expression of a subset of drought-tolerance-related genes, which were usually low under normal conditions.

## 4. Discussion

Drought stress induces a wide range of responses in plants, from molecular expression, biochemical metabolism, to physiological level [8,9]. Diverse genes and pathways are involved in these responses and plants’ adaptation to drought stress. In our study, RNA-seq technology was used to generate the de novo assembled transcriptomes of diploid and tetraploid *M. lutarioriparius*, respectively. By comparing the differential gene expression patterns in response to drought stress between diploid and tetraploid *M. lutarioriparius*, we evaluated the adaptation strategies of diploid and tetraploid *M. lutarioriparius* to drought stress and identified the candidate genes that may be used to improve the drought tolerance of plants.

Under the drought stress condition, plants avoid dehydration of cells and tissues through accumulating solutes and changing the properties of the cell wall, and use protective proteins and regulatory mechanisms to adapt to the decrease in water content [8,9]. In our study, the number of DEGs in response to drought stress in diploid *M. lutarioriparius* were much larger than those in tetraploid *M. lutarioriparius* (Figure 1), which suggested diploid *M. lutarioriparius* were more severely affected by drought stress than tetraploid *M. lutarioriparius*. Among the up-regulated genes in the leaves of diploids under drought stress, some of them showed a high, similar expression pattern between the treatment and control groups in the leaves of tetraploids (Figure 2A). Most of the up-regulated genes in leaves of tetraploid were also up-regulated in the leaves of diploids (Figure 2C). The results together suggested that diploid and tetraploid *M. lutarioriparius* might share some similar drought resistance genes.

We quantitatively and qualitatively analyzed the difference of responses to drought stress between diploid and tetraploid *M. lutarioriparius*. Quantitatively, the number of DEGs in diploids in response to drought stress was larger and the genetic effect was stronger (Figure 1). Qualitatively, under drought stress, tetraploid *M. lutarioriparius* specifically induces expression of genes directly involved in the process of drought stress tolerance. First of all, the relative abundance of key drought-tolerance-related functional categories and pathways in tetraploid *M. lutarioriparius* were higher than those in *M. lutarioriparius* (Figure 3 and Figure 4). We observed that 672 genes were up-regulated in 329 tetraploid leaves, of which 23 genes were significantly enriched in three drought-tolerance-related pathways, and 1010 genes were up-regulated in roots, of which 35 genes were significantly enriched in four drought-tolerance-related pathways (Figure 5). However, although there are many more genes induced in the leaves and roots of M313 (Figure S5), no one drought-tolerance-related pathway was enriched (Figure 5). Therefore, fewer genes were induced in the tetraploid *M. lutarioriparius*, but more drought-tolerance-related pathways were activated, while most of the up-regulated genes in the diploid *M. lutarioriparius* were passively induced.

Genes involved in ROS scavenging, biosynthesis of osmolytes, water transport, and stress-related transcription factors were generally up-regulated in diploid *M. lutarioriparius* leaves and constitutively expressed in tetraploid *M. lutarioriparius* leaves (Figure 6). The expression level of these genes in the leaves of tetraploid *M. lutarioriparius* was higher than that in the leaves of diploid *M. lutarioriparius* in control conditions (Figure 6). As tetraploid *M. lutarioriparius* is more drought-tolerant than diploid *M. lutarioriparius*, we reasoned that under normal growth condition, tetraploid *M. lutarioriparius* preadapts to drought stress through constitutive expression of a series of drought-tolerance-related genes, while diploid *M. lutarioriparius* alleviates drought stress by inducing expression of only some drought-tolerance-related genes. In conclusion, our results suggest that, on the one hand, the drought-tolerant tetraploid *M. lutarioriparius* can preadapt to drought by constitutive overexpression of a series of drought tolerance genes; on the other hand, it can adapt to drought by strongly activating other drought-related pathways.

## Figures and Tables

**Table 1:** Summary of de novo assembled transcriptomes.

	Total Unigenes	N50 (bp)	Percent GC (%)	Average Mapped Ratio (%)
M016	84,329	1211	50.07	89.70
M313	95,974	1092	48.85	88.29
Average	90,152	1152	49.46	89

## Data Availability

The RNA-seq data have been deposited in the NCBI database under BioProject number PRJNA719635.

## Supplementary Materials

The following supporting information can be downloaded at: https://www.mdpi.com/article/10.3390/genes13050873/s1. Table S1. Statistics of DEGs with orthologous transcripts in leaves and roots of two accessions. Figure S1. The phenotype of diploid and tetraploid *M. lutarioriparius* after 28 days of drought treatment. Figure S2. Length distribution of unigenes from the assembled transcriptomes in diploid and tetraploid *M. lutarioriparius*. Figure S3. The correlation of gene expression level between two biological repeats in the drought treatment group. Figure S4. Heatmap of DEGs with orthologous transcripts in the roots of diploid and tetraploid *M. lutarioriparius*. Figure S5. The number of DEGs in tetraploid and diploid. Figure S6. Heatmap of drought-tolerance-related genes in roots of diploid and tetraploid *M. lutarioriparius*.
