# Supplementary material for: Comparative Analysis of Transcriptomes of Diploid and Tetraploid Miscanthus lutarioriparius under Drought Stress
Source: Genes (Basel). 2022 May 13;13(5):873. doi: 10.3390/genes13050873 (PMC9141248; doi:10.3390/genes13050873)
Supplement: Supplementary file 1 [file genes-13-00873-s001.zip › genes-1684650-supplementary.pdf]

Supplementary Materials

Table S1. Statistics of DEGs with orthologous transcripts in leaves and roots of two accessions.

	**M313**	**M016**
up (leaves)	3,335a	672a
	1,840b	244b
up (roots)	3,200a	1,010a
	1,323b	350b
down (leaves)	11,067a	478a
	764b	237b
down (roots)	6,855a	1,380a
	2,475b	240b

The letters a represents the DEGs in M. lutarioriparius with different ploidy level, and the letters b represents the DEGs with orthologs in M. lutarioriparius with different ploidy level.

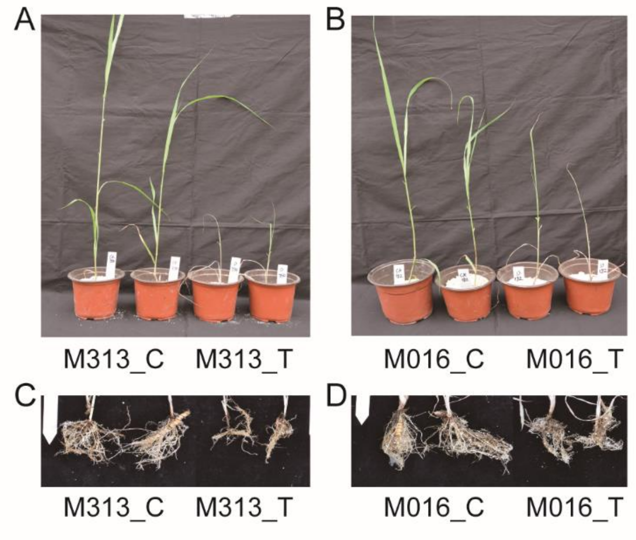

Figure S1. The phenotype of diploid and tetraploid *M. lutarioriparius* after 28 days of drought treatment. (A, C) The shoots (A) and lateral roots (C) of diploid *M. lutarioriparius* after drought-treatment after 28 days. (B, D) The shoots (B) and lateral roots (D) of tetraploid *M. lutarioriparius* after 28 days of drought-treatment. M313 represents the diploid *M. lutarioriparius*, M016 represents the tetraploid *M. lutarioriparius*. The letter C and T represent the control group and the drought treatment group, respectively.

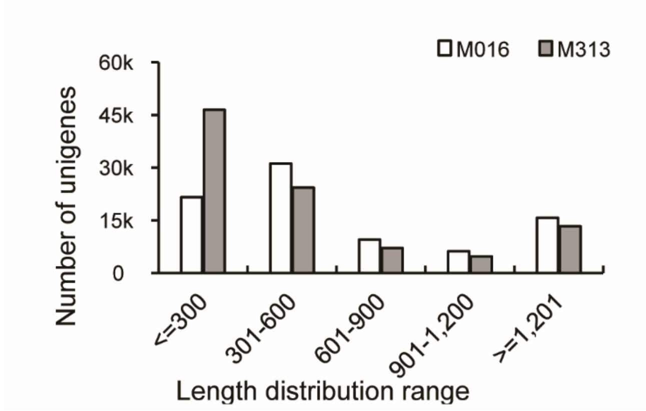

Figure. S2. Length distribution of unigenes from the assembled transcriptomes in diploid and tetraploid *M. lutarioriparius*.

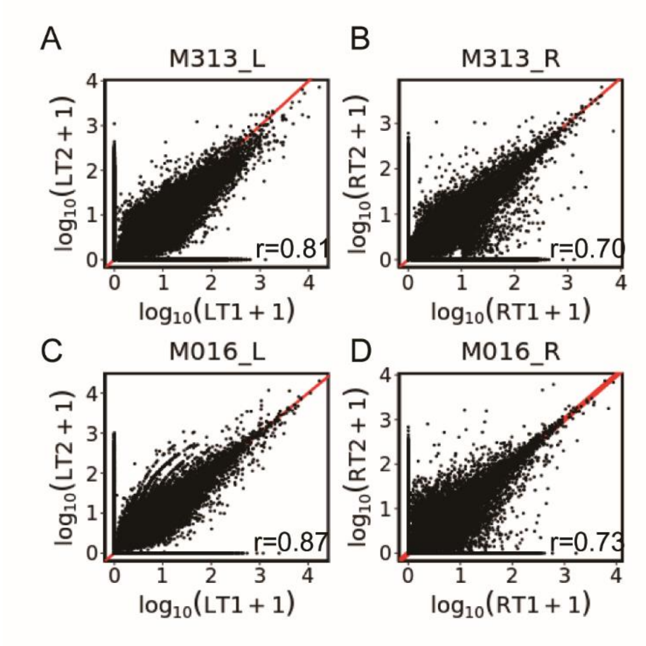

Figure. S3. The correlation of gene expression level between two biological repeats in the drought-treatment group. (A,B) The scatterplot between two biological repeats in leaves (A) and roots (B) of diploid *M. lutarioriparius*. (C,D) The scatterplot between two biological repeats in leaves (C) and roots (D) of tetraploid *M. lutarioriparius*. LT represents TPM of each transcript in leaves of treatment group and RT represents TPM of each transcript in roots of treatment group. Two biological repeats are represented by LT1 and LT2.

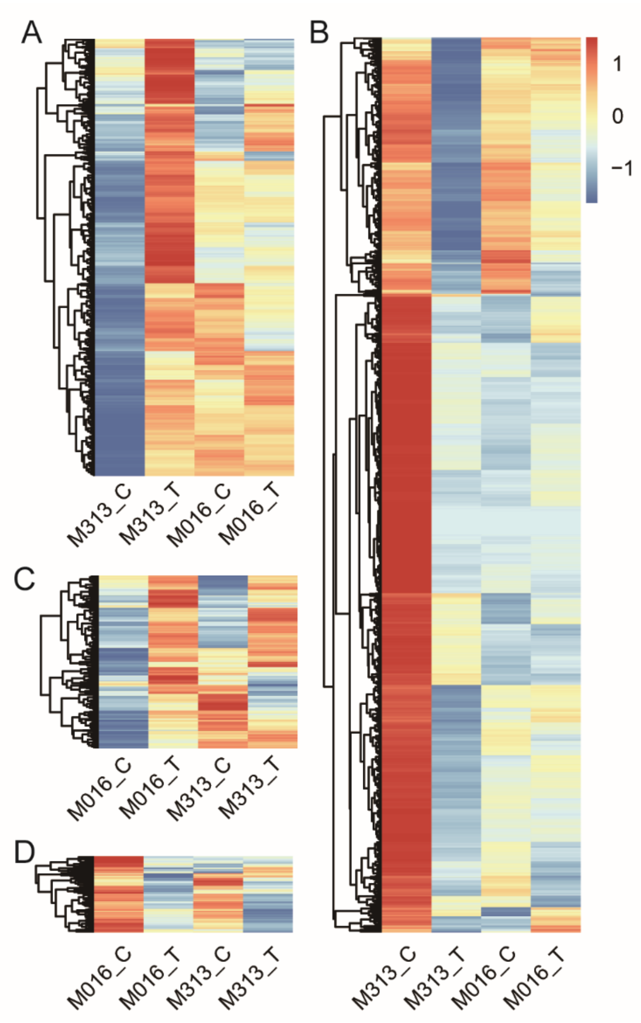

Figure. S4. Heatmap of DEGs with orthologous transcripts in the roots of diploid and tetraploid *M. lutari-oriparius*. (A) Heatmap of up-regulated transcripts with orthologs in roots of M313. (B) Heatmap of down-regulated transcripts with orthologs in roots of M313. (C) Heatmap of up-regulated transcripts with orthologs in roots of M016. (D) Heatmap of down-regulated transcripts with orthologs in roots of M016. Red and blue colors represent up-regulated and down-regulated transcripts, respectively. The darker the color is, the greater the difference of genes expression level is.

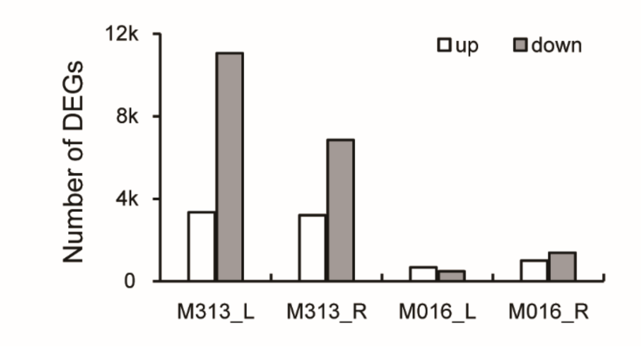

Figure. S5. The number of DEGs in tetraploid and diploid.

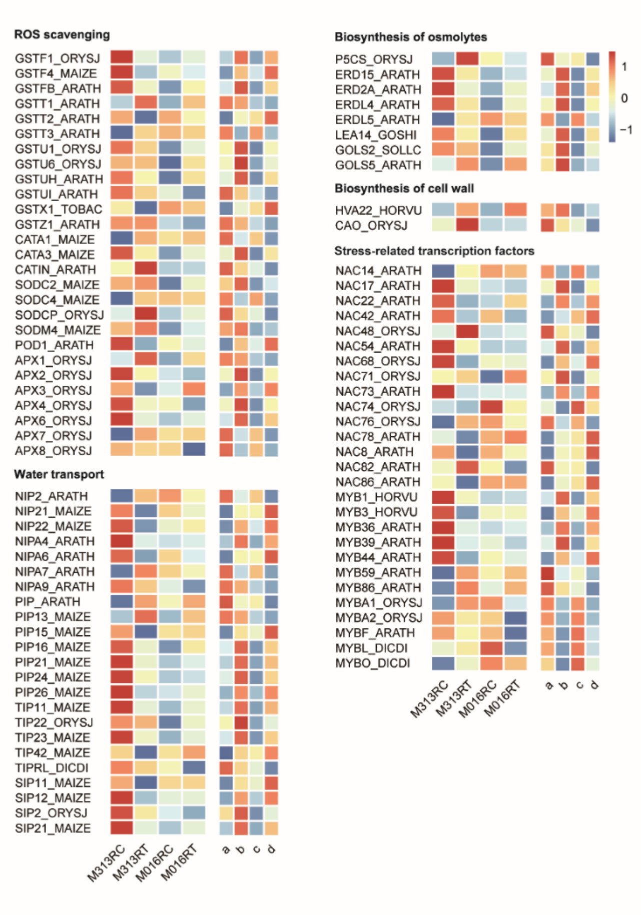

Figure. S6. Heatmap of drought tolerance-related genes in roots of diploid and tetraploid *M. lutarioriparius*. Red and blue colors represent up-regulated and down-regulated transcripts, respectively. The darker the color is, the greater the difference of genes expression level is. a, M313T vs M313C; b, M016T vs M016C; c, M016C vs M313C; d, M016T vs M313T.
